# Cardiometabolic Risk in First Episode Psychosis Patients

**DOI:** 10.3389/fendo.2020.564240

**Published:** 2020-11-24

**Authors:** Jo Smith, Lisa A. Griffiths, Marie Band, Dominic Horne

**Affiliations:** ^1^ School of Allied Health and Community, University of Worcester, Worcester, United Kingdom; ^2^ Department of Nutrition, Food and Exercise Science, Florida State University, Tallahassee, FL, United States; ^3^ Moorfields Eye Hospital NHS Foundation Trust, National Health Service, London, United Kingdom

**Keywords:** first episode psychosis, cardiometabolic risk, duration of untreated psychosis, lifestyle behaviors, metabolic syndrome, serious mental illness

## Abstract

Previous research in patients with schizophrenia in European and USA population groups has demonstrated a high prevalence of metabolic syndrome and disease progression (~35%–40%) and increased risk for cardiovascular disease and long-term mortality. Limited research has determined the prevalence of existing cardiometabolic risk factors at onset of a first episode psychosis. This cross-sectional study presents a clinical overview of the cardiometabolic risk profile in young people with first episode psychosis in the UK. Forty-six participants (72% male) clinically diagnosed with first episode psychosis (n = 25), schizophrenia (n = 13), bipolar disorder (n = 4), unspecified non-organic psychosis (n = 2) or acute psychotic episode (n = 2) with < 6 months Duration of Untreated Psychosis (DUP; mean 33.4 ± 37.2 days) were assessed for anthropometric, health risk behaviors and clinical measurements including resting heart rate, blood pressure, blood lipids, glycated hemoglobin, and prolactin. Overall, participants (aged 18–37 years) had a high prevalence of cardiometabolic risk factors due to: elevated values for BMI (73%) and abdominal adiposity (50%), blood pressure (47% prehypertensive; 23% hypertensive), resting heart rate (43%); hypercholesterolemia (32%); suboptimal HDL-C levels (36%); and hypertriglyceridemia (40%). Participants also self-reported poor health risk habits including smoking (55%), alcohol use (39%), substance use (18%), poor diet (52%), and sedentary behavior (29%). Young people with psychosis are at increased risk for cardiometabolic disorders due to elevated clinical markers and health risk behaviors. Physical health interventions (including health behavior advice) are needed early in the treatment process to address this increased risk for cardiometabolic disorders in individuals recently diagnosed with psychosis.

## Introduction

Research in people with schizophrenia, bipolar disorder and psychosis in USA and Europe has demonstrated a high prevalence of metabolic syndrome (MetS) and disease progression (~35%–40%) contributing to an increased risk for cardiometabolic disorders and long-term mortality (1–4). Evidence suggests increased risk of cardiovascular mortality is ~2.5 times higher in individuals with psychosis compared to age-matched counterparts (5–8). Patients with serious mental illness (SMI) have an increased prevalence of developing obesity, hyperglycaemia, dyslipidaemia, hypertension, hyperprolactinemia and MetS (1, 9–12). These individuals are also four to five times more likely to be smokers (13) with elevated rates of obesity up to twice those of the general population (14).

Individuals with a first episode psychosis (FEP) are particularly susceptible to rapid and pronounced weight gain (15). Antipsychotic medications, particularly obesogenic agents such as Olanzapine and Clozapine, can exacerbate weight gain, and increase levels of blood glucose and fasting triglycerides (16–18). However, Zhang et al. (19) showed glucose disturbances in first-episode drug-naïve schizophrenia patients, suggesting that medication is not the sole contributor leading to these adverse outcomes but may combine with underlying psychopathology factors, notably, impaired glucose homeostasis (20), glucose tolerance, and insulin resistance (21). Stahl et al. (22) reported that even without weight gain, increased fasting triglycerides strongly correlates with development of insulin resistance. This is consistent with recent research demonstrating higher incidences of type 2 diabetes mellitus (T2DM) in individuals taking Olanzapine, Clozapine, and some other antipsychotic medications (23, 24). In comparison, the general population has benefited from improved cardiovascular health due to primary and secondary prevention, while SMI patients remain at increased risk due to less opportunity for cardiovascular risk screening, less access to general health care, sedentary behavior, weight gain, and poor diet (25).

Research in the USA and UK has demonstrated a high prevalence of metabolic syndrome (MetS) in schizophrenia patients (~13%–57%) (1, 10) which presents an increased risk for cardiovascular disease (CVD), impaired daily functioning and long-term mortality (18–26). There is limited research in European populations comparing the metabolic risk profile of individuals with early psychosis compared to age-matched general population counterparts (27, 28). In a UK context, there have been two studies that have investigated cardiometabolic risk in first episode patients. The first paper by Ryan et al. (29) examined the prevalence of fasting glucose tolerance in hospitalized first episode patients with schizophrenia. The second paper was a large scale, prospective cohort study of an ethnically diverse group of patients with FEP, aged 16–65 years, within 6 months of their first presentation with psychosis (30) whose cardiometabolic outcomes were monitored over a 12-month period. Neither study specified the length of time from the onset of psychotic illness to initiation of antipsychotic treatment of study participants, known as the duration of untreated psychosis (DUP). A measure of DUP is needed to control for the influence of duration of psychotic illness and the development of illness-related health risk habits such as poor diet, decreased physical activity, and smoking on specific cardiometabolic measures, particularly glucose homeostasis (31) and risk for MetS (32). This is the first UK study to look at cardiometabolic risk in association with health risk behaviors in an unselected, young, early psychosis cohort, within 3 months of psychosis onset. The aim of this cross-sectional study is to present a clinical overview of the cardiometabolic risk prevalence for individuals with FEP with a short DUP on presentation to a UK Early Intervention in Psychosis (EIP) service.

## Materials and Methods

### Patients and Diagnoses

Clinical data collection for FEP patients was conducted in the first six weeks following acceptance onto caseload of two UK Early Intervention in Psychosis (EIP) teams, between June 2014 to June 2015. The EIP service remit is to work with young people (14–35 years) presenting with FEP or bipolar disorder. Forty-six participants (~68% of annual clinical intake) clinically diagnosed with a provisional diagnosis of FEP (33), schizophrenia, bipolar disorder, acute psychotic episode, and unspecified non-organic psychosis with a DUP < 6 months were included in the study (see **Table 1**). Clinical diagnosis was determined by an independent consultant psychiatrist during routine assessment using International Classification of Diseases (ICD)-10 (34) criteria for a psychotic disorder.

**Table 1:** Demographics, illness, and treatment characteristics by sex.

Characteristic	Total(n = 46)	Males(n = 33)	Females(n =13)
Age (y)	24.5 (4.4)	24.0 (3.9)	25.7 (5.4)
**Duration Since Onset**			
0–3 mo	40 (87.0%)	29 (87.2%)	11 (84.6%)
3–6 mo	6 (13%)	4 (12.1%)	2 (15.4%)
**Ethnicity (%)**			
White	40 (87.0%)	28 (84.8%)	12 (92.3%)
Asian	4 (8.8%)	4 (12.1%)	0
Chinese	1 (2.2%)	0	1 (7.7%)
Undeclared	1 (2.2%)	1 (3.0%)	0
**Primary SCID Diagnosis**			
FEP_a_	25 (54.3%)	19 (57.6%)	6 (46.2%)
Schizophrenia	13 (28.3%)	10 (30.3%)	3 (23.1%)
Bipolar	4 (8.7%)	1 (3.0%)	3 (23.1%)
Acute psychotic episode	2 (4.3%)	2 (6.1%)	0
Non-organic	1 (2.2%)	0	1 (7.7%)
Other	1 (2.2%)	1 (3.0%)	0
**Antipsychotic Treatment (%)**			
No medication	9 (17.8%)	7 (21.1%)	2 (15.4%)
Aripiprazole	4 (8.9%)	4 (12.1%)	0
Clozapine	3 (6.7%)	3 (9.1%)	0
Olanzapine	13 (28.9%)	9 (27.3%)	4 (30.8%)
Paliperidone	1 (2.2%)	1 (3.1%)	0
Quetiapine	13 (28.9%)	7 (21.2%)	6 (46.2%)
Risperidone	2 (4.4%)	1 (3.0%)	1 (7.7%)
Other	1 (2.2%)	1 (3.1%)	0
**Antidepressant Treatment (%)**			
No medication	31 (67.4%)	25 (75.8%)	6 (46.2%)
Citalopram	1 (2.2%)	1 (3.0%)	0
Sertraline	2 (4.3%)	2 (6.1%)	0
Venlafaxine	4 (8.7%)	2 (6.1%)	2 (15.4%)
Fluoxetine	2 (4.3%)	0	2 (15.4%)
Other	5 (10.9%)	3 (9.1%)	3 (23.1%)

^a^provisional diagnosis of First Episode of Psychosis (FEP) (33).

Participation eligibility was determined by an EIP mental health nurse. Exclusion criteria included: patient too unwell or declined to participate in a physical health assessment, early discharge, or where care involvement was limited (i.e., studying/working elsewhere). The NHS Health Research Authority decision making process classified this project as a clinical service evaluation. Ethical approval was obtained from the University Research Ethics Committee (REC approval no.:UWEC2014JS1).

### Clinical Assessment

Upon caseload acceptance, participants had a routine physical health assessment conducted by a mental health nurse. Clinical markers included DUP, medical history, medications and health risk behaviors. Physical health markers were assessed against World Health Organization (35), International Diabetes Federation (IDF) criteria for MetS (36), and Lester UK Adaptation (37) criteria to determine risk levels for cardiovascular and metabolic disorders. Measures included: body mass index (BMI), waist circumference, resting heart rate and blood pressure, glycated hemoglobin (HbA1c), fasting plasma glucose (FPG), and blood lipids. Blood lipids included measures of total cholesterol, low-density lipoprotein (LDL-C), high-density lipoprotein (HDL-C), and triglycerides. Prolactin levels were assessed using criteria published in Taylor et al. (38). Abdominal adiposity was assessed using waist circumference where measurements were made at the approximate midpoint between the lower margin of the last palpable rib and the top of the iliac crest (35). Blood tests to determine HbA1c and/or FPG, blood lipids and prolactin were requested by the patient’s general practitioners and conducted by NHS pathology departments. All blood data were collected from electronic patient records.

Health risk behaviors were assessed using self-reported measures for diet [eating > 5 fruit/vegetables per day; equivalent to 400 g a day based on 80 g portions; (39)], tobacco use (current smoker or within last 6 months), alcohol use [Alcohol Use Disorders Identification Test; (40)], substance use (yes/no response), and PA levels [Exercise Vital Sign (EVS); (41)]. Sedentary behaviors were determined as engaging in PA less than 90 min of moderate PA per week (42).

### Data Analysis

We conducted a cross-sectional analysis of baseline clinical assessment data. Frequency analyses were conducted to determine demographic data, and prevalence of cardiometabolic and health risk factors (SPSS v. 25.0). Independent sample t-tests were performed on scaled data and Mann-Whitney U tests were performed on categorical data to determine sex differences across outcome measures. A binary logistic regression analysis was performed to ascertain impact of health risk behaviors, DUP and antipsychotic medications on risk of dyslipidemia, MetS, and hypertension. Scaled data were presented as mean (SD). Categorical data was presented as frequency and/or percent of the total population. Alpha level was set at *p* < 0.05.

## Results

### Demographic, Illness, and Treatment Characteristics

Participants included 46 patients (72% males; 28% females), primarily White British, clinically diagnosed with FEP with a DUP < 3 months (mean DUP 33.4 ± 37.2 days); 63% had a DUP < 1 month (see **Table 1**). Physical health assessments were conducted in the first six weeks following acceptance onto the EIP team caseload. At assessment, 82% were in receipt of antipsychotic medication, 33% were on antidepressant medication, and no patients were prescribed mood stabilizers.

Forty-one participants (89%) completed all physical measurements; only 20 participants (44%) completed all blood profile assessments. Reasons for incomplete assessments included: differences in blood tests requested by general practitioner (LDL and triglycerides were not ordered in one service), refusal to give blood and/or participate in sensitive physical measurements (e.g., waist circumference), declined based on religious concerns, repeat non-attendance for screening appointments, or no reason given.

### Cardiometabolic Risk Factors

**Table 2** provides data for cardiometabolic risk status and treatment for the total population sample and by sex.

**Table 2:** Cardiometabolic risk status at baseline by sex.

Variable	Threshold	N	Total	N	Males	N	Females	*p* Value
Age		46	24.5 (4.4)	33	24.0 (3.9)	13	25.7 (5.4)	0.252
**Body Composition**								
Weight (kg)		44	87.8 (16.6)	32	87.4 (14.8)	12	89.6 (21.5)	0.668
BMI (kg.m^2^)	>25.0	44	28.9 (5.8)	32	27.7 (5.0)	12	31.9 (6.9)	0.032*
Weight Status (%)								
*Underweight*	>18.5 kg.m^2^	44	2 (4.5%)	32	2 (6.3%)	12	0	–
*Normal*	18.5–24.9 kg.m^2^	44	10 (22.7%)	32	9 (28.1%)	12	1 (8.3%)	–
*Overweight*	25.0–29.9 kg.m^2^	44	16 (36.4%)	32	11 (34.4%)	12	5 (41.7%)	–
*Obese*	30.0–34.9 kg.m^2^	44	11 (25.0%)	32	9 (28.1%)	12	2 (16.7%)	–
*Morbidly Obese*	>35.0 kg.m^2^	44	5 (11.4%)	32	1 (3.1%)	12	4 (33.3%)	–
Waist Circumference (cm)		42	95.1 (15.9)	30	94.7 (15.3)	12	96.2 (18.1)	0.781
* IDF: Acceptable range* * WHO: Acceptable range*	♂ < 94 ♀ < 80	42	17 (40.5%)	30	16 (53.3%)	12	1 (8.3%)	–
* IDF: Elevated WC* * WHO: Increased Risk*	♂ > 94 ♀ > 80	42	4 (9.5%)	30	2 (6.6%)	12	2 (16.6%)	–
* WHO: Substantially* *increased Risk*	♂ > 102 ♀ > 88	42	21 (50%)	30	12 (40.0%)	12	9 (75.0%)	–
**Cardiometabolic Measurements**								
SBP (mm Hg)		43	126.8 (19.8)	32	131.3 (20.0)	11	113.8 (12.5)	0.010*
DBP (mm Hg)		43	80.7 (16.7)	32	82.4 (17.9)	11	75.5 (11.6)	0.242
*WHO Prehypertensive (%)*	SBP: 120–139 mmHg DBP: 80–89 mm Hg	43	20 (46.5%)	32	12 (37.5%)	11	8 (72.7%)	–
*WHO Hypertension (%)*	>140/90 mm Hg	43	10 (23.3%)	32	10 (31.3%)	11	0	–
*IDF Hypertension (%)*	>130/85	43	20 (46.5%)	32	17 (53.1%)	11	3 (27.3%)	0.143
Resting pulse (beats.min^−1^)	>80	42	78.7 (17.6)	31	79.4 (18.0)	11	76.7 (16.9)	0.672
Total Cholesterol (mmol.L^−1^)	≥5.2	43	4.7 (1.1)	31	4.9 (1.2)	12	4.5 (0.8)	0.328
LDL Cholesterol (mmol.L^−1^)		18	3.0 (0.8)	13	3.0 (0.7)	5	2.8 (1.0)	0.532
HDL-C (mmol.L^−1^)	♂ <1.03 ♀ <1.29	42	1.3 (0.3)	31	1.2 (0.3)	11	1.3 (0.4)	0.262
Triglycerides (mmol.L^−1^)	>1.7	20	1.6 (1.0)	14	1.8 (1.1)	6	1.2 (0.7)	0.257
Dyslipidemia (%)	number (%) > 1 lipid value above recommended level	43	25 (58.1%)	31	19 (61.3%)	12	6 (50.0%)	–
FPG (mmol.L^−1^)	>5.6	32	5.1 (0.8)	21	5.1 (0.9)	11	5.0 (0.5)	0.693
HbA1c (mmol.mol)	>42	31	36.7 (7.8)	22	37.5 (8.9)	9	34.6 (3.4)	0.343
MetS	number (%) that met IDF criteria	46	14 (30.4%)	33	11 (33.3%)	13	3 (23.1%)	–
Prolactin (mIU/L^−1^)	♂ < 424 ♀ < 530	37	501.4 (710.9)	26	295.2 (172.6)	11	988.7 (1172.0)	0.079
**Health Risk Behaviors**								
Smoking	Currently smoke or within past 3 mos.	44	24 (54.5%)	31	19 (61.3%)	13	5 (38.5%)	0.110
Alcohol Use	Any alcohol use	44	17 (38.6%)	31	11 (35.5%)	13	6 (46.2%)	0.512
Substance Use	Any recreational drug use	44	8 (18.2%)	31	6 (19.4%)	13	2 (15.4%)	0.758
Unhealthy eating	< 5 fruits/veg.d^−1^	44	23 (52.3%)	31	13 (41.9%)	13	10 (76.9%)	0.036*
Sedentary Lifestyle	< 90 min.wk^−1^	44	17 (28.6%)	31	12 (38.7%)	13	5 (38.5%)	0.988

*p < 0.05.

BMI, body mass index; IDF, International Diabetes Federation; WHO, World Health Organization; WC, waist circumference; SBP, systolic blood pressure; DBP, diastolic blood pressure; HDL–C, high density lipoprotein cholesterol; HbA1c, glycated haemoglobin; MetS, metabolic syndrome.

#### Body Composition

Males and females had similar mean body mass and waist circumference; females’ mean BMI was 14% higher compared to males’ (*p* = 0.03). Some participants declined body mass (n = 2) and waist circumference (n = 4) measures.

According to WHO BMI thresholds, 73% of participants were overweight or obese. For abdominal adiposity, 9.5% of participants met IDF criteria for *elevated risk* and WHO criteria for *increased risk* of metabolic complications and 50% met WHO criteria for a *substantially increased* *risk* of metabolic complications.

### Cardiometabolic Measurements

Forty-five percent had a resting pulse > 80 beats.min^−1^; 21% had a resting pulse > 100 beats.min^−1^. Two-thirds of the cohort was classified as pre-hypertensive (47%) or hypertensive (23%) using WHO thresholds compared to 47% having hypertension using IDF criteria. One participant was being treated with antihypertensive medication; three participants declined to be tested. No females met the WHO threshold for hypertension whereas three females met the IDF criteria. Males had ~14% higher systolic blood pressure (SBP) compared to females (*p* = 0.01); no sex differences were observed for DBP or resting pulse.

Fifty-eight percent had one or more clinical values which met WHO and IDF criteria for lipid disturbance: hypercholesterolemia (32%), suboptimal HDL-C levels (36%), and hypertriglyceridemia (40%). Males and females had similar mean values for blood lipid assessments and prevalence rates for dyslipidemia. Depending on EIP team location, glucose metabolism was determined using either FPG and/or HbA1c. According to IDF, 22% met the criteria for raised FPG and 30% for MetS. Using Lester UK Adaptation thresholds, 24% were identified as being at high risk for T2DM: 22% had elevated FPG, 6% had raised HbA1c and one participant was previously diagnosed with T2DM. Ninety-six percent of patients had at least one of the Lester UK high risk thresholds. Four individuals declined to have blood lipid and glucose measures assessed. Only 37 participants had prolactin measured, where four males (15%) and six females (55%) exceeded the prolactin threshold for hyperprolactinemia.

Across all participants, significant relationships were observed for waist circumference and resting pulse (*r* = 0.37; *p* = 0.02), SBP (*r* = 0.35; *p* = 0.03), diastolic blood pressure (DBP) (*r* = 0.32; *p* = 0.04), and HDL-C (*r* = −0.38; *p* = 0.02). Resting pulse was correlated to DBP (*r* = 0.510; *p* < 0.001) and HbA1c (*r* = 0.47; *p* = 0.01). HbA1c also showed a medium correlation to HDL-C (*r* = 0.45; *p* = 0.01). In males, waist circumference was positively correlated to body mass (*r* = 0.93*, p* < 0.001), resting pulse (*r* = 0.39, *p* = 0.04), and total cholesterol (*r* = 0.42, *p* = 0.03), and inversely correlated to HDL-C levels (*r* = −0.48, *p* = 0.01). In females, only waist circumference and body mass had a positive correlation (*r* = 0.62, *p* = 0.04). No other relationships were observed between anthropometric and cardiometabolic markers (*p* > 0.05).

### Effect of Medications and DUP on Risk Profile

Of the 16 individuals on high risk obesogenic medication, Clozapine (n = 3) or Olanzapine (n = 13), 81% were overweight/obese, 56% had elevated abdominal obesity, 38% were pre-hypertensive and 38% hypertensive according to WHO guidelines. Using IDF guidelines, 56% were hypertensive, 69% had dyslipidaemia, and 31% met the criteria for MetS; three participants declined blood lipid measurements.

Twenty-nine participants had a DUP <1 month (age range 19–32 years). Even with a short DUP, 18% met WHO and 43% met IDF criteria for high blood pressure, 32% had elevated cholesterol, 39% had sub-optimal HDL-C, 33% had high triglycerides, 54% met the IDF threshold for dyslipidemia, 35% met criteria for MetS, and 93% had at least one of the Lester UK high risk thresholds (see **Table 3**). Binary regression analysis revealed no significant relationships (p > 0.05) where neither antipsychotic medication or DUP nor their interaction, were able to predict the likelihood of MetS, dyslipidemia or hypertension in this cohort.

**Table 3:** Cardiometabolic risk status for individuals with DUP < 1 month.

Variable	Threshold	N	Total	N	Males	N	Females	*p* Value
Age		29	24.6 (3.9)	20	24.2 (3.6)	9	25.7 (4.6)	0.362
**Body Composition**									
Weight (kg)		29	83.8 (14.2)	20	84.1 (14.4)	9	83.3 (14.8)	0.862
BMI (kg.m^2^)	>25.0	29	27.8 (5.8)	20	26.7 (5.4)	9	30.1 (6.1)	0.141
Weight Status (%)								
*Underweight*	>18.5 kg.m^2^	29	2 (6.9%)	20	2 (10.0%)	9	0	–
*Normal*	18.5–24.9 kg.m^2^	29	8 (27.6%)	20	7 (35.0%)	9	1 (11.1%)	–
*Overweight*	25.0–29.9 kg.m^2^	29	10 (34.5%)	20	5 (25.0%)	9	5 (55.6%)	–
*Obese*	30.0–34.9 kg.m^2^	29	6 (20.7%)	20	5 (25.0%)	9	1 (11.1%)	–
*Morbidly Obese*	>35.0 kg.m^2^	29	3 (10.3%)	20	1 (5.0%)	9	2 (22.2%)	–
Waist Circumference (cm)		27	93.7 (14.1)	19	92.5 (14.4)	8	96.8 (13.7)	0.478
*IDF: Acceptable Range* * WHO Acceptable Range*	♂ < 94 ♀ < 80	27	10 (37.0%)	19	10 (52.6%)	8	0	
* IDF: Elevated WC* *WHO: Increased Risk*	♂ > 94 ♀ > 80	27	3 (11.1%)	19	1 (5.3%)	8	2 (25.0%)	–
*WHO: Substantially* *increased Risk*	♂ > 102 ♀ > 88	27	14 (51.9%)	19	8 (42.1%)	8	6 (75.0%)	–
**Cardiometabolic Measurements**									
SBP (mm Hg)		28	125.8 (17.6)	20	130.2 (17.6)	8	115.0 (13.0)	0.037*
DBP (mm Hg)		28	84.4 (18.6)	20	86.9 (20.8)	8	78.0 (9.7)	0.260
*WHO Prehypertensive (%)*	SBP: 120–139 mmHg DBP: 80–89 mm Hg	28	16 (57.1%)	20	9 (45.0%)	8	7 (87.5%)	–
*WHO Hypertension (%)*	>140/90 mm Hg	28	5 (17.9%)	20	5 (25.0%)	8	0	–
*IDF Hypertension (%)*	>130/85	28	12 (42.9%)	20	10 (50.0%)	8	2 (25.0%)	0.328
Resting pulse (beats.min^−1^)	>80	27	79.3 (17.8)	19	79.6 (18.4)	8	78.6 (17.4)	0.902
Total Cholesterol (mmol. L^−1^)	≥5.0	26	4.5 (0.9)	18	4.4 (0.9)	8	4.5 (0.8)	0.754
LDL-C (mmol. L^−1^)		12	2.7 (0.8)	8	2.7 (0.6)	4	2.8 (1.1)	0.821
HDL-C (mmol. L^−1^)	♂ < 1.03 ♀ < 1.29	26	1.2 (0.3)	18	1.2 (0.3)	8	1.2 (0.3)	0.796
Triglyceride (mmol. L^−1^)	> 1.7	12	1.7 (1.1)	8	1.8 (1.3)	4	1.3 (0.8)	0.508
Dyslipidaemia (%)	number (%) > 1 lipid value above recommended level	26	14 (53.8%)	18	9 (50.0%)	8	5 (62.5%)	0.644
FPG (mmol. L^−1^)	>5.6	20	5.0 (0.7)	12	4.9 (0.8)	8	5.1 (0.5)	0.589
HbA1c (mmol.mol)	>42 (6.0%)	18	37.6 (10.0)	11	39.9 (12.3)	7	34.0 (2.6)	0.233
MetS	number (%) that met IDF criteria	29	10 (34.5%)	20	7 (35.0%)	9	3 (33.1%)	0.945
Prolactin (mIU/L^−1^)	♂ < 424 ♀ < 530	22	676.5 (883.7)	14	332.9 (218.3)	8	1277.6 (1267.0)	0.074
**Health Risk Behaviors**									
Smoking	Currently smoke or within past 6 months.	28	17 (60.7%)	19	13 (68.4%)	9	4 (44.4%)	0.174
Alcohol Use	Any alcohol use	28	12 (42.9%)	19	8 (42.1%)	9	4 (44.4%)	0.909
Substance Use	Any recreational drug use	28	4 (14.3%)	19	3 (15.8%)	9	1 (11.1%)	0.746
Unhealthy eating	<5 fruits/veg.d^−1^	28	15 (53.6%)	19	8 (42.1%)	9	7 (77.8%)	0.083
Sedentary Lifestyle	<90 min.wk^−1^	28	11 (39.3%)	19	8 (42.1%)	9	3 (33.3%)	0.663

*p < 0.05.

BMI, body mass index; IDF, International Diabetes Federation; WHO, World Health Organization; WC, waist circumference; SBP, systolic blood pressure; DBP, diastolic blood pressure; HDL–C, high density lipoprotein cholesterol; HbA1c, glycated haemoglobin; MetS, metabolic syndrome.

### Health Risk Factors

Participants’ self-reported health risk behaviors including smoking (55%), alcohol intake (39%), substance use (18%), poor diet (52%), and sedentary behavior (29%). Fifty-five percent reported smoking within the last 3 months with 46% smoking < 10 cig.d^−1^. No patients reported receiving smoking cessation support (e.g., nicotine patches) nor did clinical notes indicate a referral for smoking cessation support. Although non-significant, males reported higher levels of smoking (61%) compared to females (39%). Thirty-nine percent of participants reported regular alcohol intake with both males (36%) and females (46%) reporting regular alcohol use. Eighteen percent of participants reported regular substance use; males and females reported similar levels of use (15%–19%). Fifty-two percent of participants reported eating five fruits/veg.d^−1^ with a higher proportion of males (58%) meeting the recommended amount compared to females (23%) (*p* = 0.04). Twenty-nine percent reported having a sedentary lifestyle with males and females reporting similar PA levels (39%).

Compared to UK age-matched peers, this cohort reported higher prevalence for high BMI, abdominal adiposity, high blood pressure, raised glucose, and smoking (see **Table 4**). Considerably higher levels of overweight/obesity, abdominal adiposity, and high blood pressure were reported in this cohort compared to Correll et al. (1) and Hahn et al. (43); differences in measuring criteria for health risk behaviors limited our ability to compare prevalence data.

**Table 4:** Comparison of cardiometabolic risk factor prevalence to age-matched peers.

Variable	This study (UK)	UK age-matched general population average (2015)_a_		Correll et al. (1)_b_ (USA)	Hahn et al., (43)_c_ (Australia)
Age (years)	16–34	16–24	25–34	15–40	18–24
Overweight/Obese	72.8%	38.8%	52.3%	48.3%	55.0%
Abdominal Adiposity	50.0%	20%	28%	17.5%	–
Pre-hypertensive_d_	46.5%	–	–	39.9%	–
Hypertension_d_	23.3%	3.2%	6.3%	10.0%	8.0%
High Total Cholesterol	40.0%	–		15.4%	12.6%
Dyslipidaemia	58.1%	–		56.5%	–
Elevated FPG	21.9%	–	–	6.9%	16.1%
Elevated HbA1c	6.4%	0.6%	0.8%	–	12.9%
MetS	30.4%	–		13.2%	–
Smoking	54.6%	24%	24%	50.8%	67.7%
Alcohol Use	38.6%	50%	58%	–	8.2%
Substance Use	18.2%	18%		–	–
Unhealthy Eating	52.3%	72%	74%	–	77.9%
Sedentary Lifestyle	38.6%	–	–	–	41.2%

_a_(43). High blood glucose = doctor diagnosed diabetes.

_b_(1). Abdominal adiposity = waist circumference > 102 cm for males and females.

_c_(42). Unhealthy eating = < 4 fruit and vegetable per day. Alcohol = > 4 drinks per day, 4 or more times per week. Sedentary lifestyle = < 2.5 hr.wk^−1^ of moderate or < 1 hr.wk^−1^ of vigorous exercise.

_d_(35). Blood pressure classifications were based on WHO guidelines.

## Discussion

The aim of this study was to present an overview of the cardiometabolic risk profile and health risk behaviors in an unselected, young, early psychosis cohort, within 3 months of their psychosis onset presenting at a UK EIP service. Participants had a high prevalence of cardiometabolic risk factors due to elevated values for BMI, waist circumference, resting pulse, blood pressure, and blood lipids. Unhealthy health risk behaviors, including smoking, sedentary lifestyle, and poor diet, may contribute to an elevated risk for CVD. This is the first UK study to demonstrate that young people with psychosis, with a short DUP and very early in EIP treatment, are at increased risk for cardiometabolic disorders. This data highlights the need for physical health and health behavior interventions early in the treatment process, particularly for individuals taking the most obesogenic medications, to address the increased risk for cardiometabolic disorders in individuals recently diagnosed with psychosis.

### Prevalence of Cardiometabolic Risk Markers

Compared to UK age-matched individuals and previous published research, this cohort had a higher prevalence of overweight/obesity, abdominal adiposity, hypertension, and elevated lipid measures (1, 3, 30, 43, 44). Despite a short DUP of less than 3 months since their psychosis onset and having a physical health assessment within 6 weeks of being taken on to EIP caseload, 93% met at least one of the Lester UK Adaptation “Red Zone” threshold criteria (37). This may, in part, be due to the high number (35%) of individuals prescribed high risk obesogenic antipsychotic medications. Females appeared at particular risk for obesity and increased abdominal obesity which may relate to the self-reported unhealthy eating, sedentary lifestyle, and antipsychotic medication prescribing.

Individuals in this younger age group are not typically considered at risk for MetS; however, 58% had dyslipidemia, 40% had hypertriglyceridemia, and 30% of participants met the IDF MetS criteria. High triglycerides, typically elevated in FEP, are a precursor for T2DM (31). The MetS prevalence in this cohort compares favourably to the elevated risk for MetS described for people with schizophrenia and related psychotic disorders identified in Vancampfort et al.’s (45) meta-analysis. Vancampfort et al. (45) reported the weighted estimated mean prevalence of MetS in population-based studies was 32.6%; 1.58 times higher risk than the general population. In young people with SMI, weight gain is exacerbated as they are more likely to engage in unhealthy health risk behaviors (46–48). It is particularly during adolescence when there is the greatest likelihood of excessive weight gain (>45 pounds) where the prevalence of obesity doubles between pre-adolescence (~17%) and young adulthood (~34%) (49).

Individuals prescribed obesogenic antipsychotic medications are at even greater risk for obesity and obesity related disorders (50). However, there is preliminary evidence that metabolic disturbances may begin early even prior to starting medication (3). Mitchell et al.’s (3) meta-analysis of unmedicated and first episode schizophrenia patients found an overall rate of MetS of approximately 10% where the metabolic risk in FEP was not significantly increased compared to unmedicated patients. However, this finding may be explained by the inclusion of only a limited number of first episode patients who had been exposed to antipsychotic medication (5 out of 26 FEP studies) and where antipsychotic exposure, in 3 of those 5 studies, was very short from less than 2 weeks to less than 3 months. Given our sample were also young and within 3 months of their psychosis onset, based on this meta-analysis, we should have expected a lower cardiometabolic risk in our cohort of first episode early psychosis patients. However, even though individuals were picked up very quickly following psychosis onset and our clinical assessments took place within 6 weeks of acceptance onto an EIP caseload, the majority (80%) were already in receipt of and established on antipsychotic medication. Information about dosage and length of exposure to antipsychotic medication were not collected in this study. This may limit our findings as these are potential confounding factors which may influence the degree of cardiometabolic risk observed in these participants.

This is one of the first UK papers which has identified that clinical markers of MetS were most evident in those treated promptly with antipsychotic medications based on DUP (<1 month). Excess abdominal adiposity in this cohort was correlated to elevated resting pulse, SBP, DBP, and lower HDL-C levels increasing the risk for metabolic and cardiovascular disorders, particularly MetS, earlier in the life course. Longer term physical health outcomes need to be considered alongside mental health outcomes where currently prompt antipsychotic treatment may be at the expense of physical health morbidity and mortality.

### Health Risk Behaviors Contributing to Increased Risk

Across the UK, smoking and alcohol use has been steadily declining (51, 52). The smoking prevalence in this sample (55%) is similar to comparable SMI populations (50%–68%) (1, 43, 53) and the 47% smoking rate observed in FEP patients in the Mitchell et al.’s (3) meta-analysis, but is considerably lower than the more recent, Gaughran et al. (30) and Lally et al. (53) studies which identified much higher rates of cigarette smoking (76.8% and 78% respectively) in FEP patients. These continued higher rates of smoking in FEP are noteworthy when compared to rates of 19%–21% in the age-matched general population where we have seen significant reductions in cigarette use across the UK (51). Substance and alcohol use were at or below national averages for UK age-matched adults (18% and 81%, respectively) (44, 52). Previous research has argued nicotine, alcohol, and substance use may be used to self-medicate to alleviate psychiatric symptoms (e.g. side effects, cognitive deficits) by increasing dopamine levels (47, 54). Manzella et al. (55) suggested there is a lack of data to support a self-medication hypothesis to explain high smoking prevalence in this population. Elevated rates of addictive substances may also be due to habit, routine, social contact, relaxation, and as a control mechanism (56, 57). As smoking has been shown to double the risk of morbidity and mortality (58), and is one of the largest potentially modifiable risk factors that is present soon after diagnosis, it is an important early target for cardiometabolic risk prevention and, as such, evidence based smoking cessation programs should be prioritized (59, 60). At the time of study, no pathway to refer for smoking cessation support was available in either primary or secondary care locally which may explain the absence of evidence in clinical notes of patients receiving nicotine patches or onward referral to smoking cessation services. Although even if a pathway had been available, people with SMI have not historically successfully engaged with generic smoking cessation services (60). The recent SCIMITAR+ study (60) successfully trained mental health practitioners to deliver a bespoke behavioral (evidence-supported techniques to change smoking behavior) combined with pharmacological smoking cessation interventions to an older group of patients with SMI (mean age 47 years).The SCIMITAR+ intervention increased engagement and chances of successful quitting at 6 months, which were more than twice the rates of those who received usual care, although these differences were not significant by 12 months. Long-term smoking cessation remains difficult to achieve and is a continuing challenge for nicotine dependence in any population, not just psychosis (60). Further research is needed to examine whether the SCIMITAR+ intervention could be adapted to be delivered in EIP services with first episode patients where smoking habits and nicotine dependence may be less strongly established and, potentially, its impact might be greater (61, 62).

Meaningful comparisons of PA and unhealthy eating were difficult as previous studies used different criteria (i.e., PA < 150 min.wk^−1^ and diet > 4 fruit/veg.d^−1^ (1, 43). High body mass and abdominal obesity are consistent with poor dietary habits and lower levels of PA. Although there are validity concerns about self-reported behavioral data (63), self-reported assessments of diet (64, 65), PA, and substance use are accepted methods to identify potential population health risks and conform to NICE Psychosis and Schizophrenia Clinical Guidelines CG178 (66) and NICE Psychosis and Schizophrenia Quality standards (QS80) (67) for health risk behaviors data collection. The use of more reliable and valid objective measures of health risk behaviors would enhance rigor, data confidence and comparability between studies but may not be practical, affordable, or even achievable in a busy, routine clinical service setting. Furthermore, this may inhibit successful screening if measures such as wearing an accelerometer or blood/urine tests are viewed as intrusive and unacceptable to this population given in this study, less than half of participants agreed to complete blood profile assessments and several refused intimate measures which fitting monitoring equipment, such as an accelerometer for measuring PA, would necessarily entail. Notwithstanding these potential measurement limitations, these combined health risk factors contribute to elevated CVD risk markers in young people with psychosis providing a rationale for health behavior intervention to systematically address unhealthy health risk behaviors.

### Evaluation of Clinical Service

This evaluation identified a number of physical health screening challenges. A key finding was inconsistent screening of physical health risk factors and inadequate recording in the clinical notes of subsequent referrals for monitoring and intervention of patients identified as being at risk. Patient challenges to obtaining complete health assessment data included refusal to give blood, refusal of taking intimate measures (i.e., waist circumference, bloods) on religious grounds, and non-attendees. UK standards require completion of all seven clinical markers to meet national EIP audit criterion (CQUIN 2015–2017) (68) which are limited by these patient challenges. Elevated values for clinical markers were not supported with documented evidence of specialist referral. Referral for specialist diabetes support was hampered by the absence of clear, referral pathways directly from mental health services and instead, relied on general practitioner referral when patients were identified as at risk and flagged up for specialist assessment and intervention. No pathway to refer for generic smoking cessation support was available in either primary or secondary care locally which meant patients who expressed a desire to stop smoking were unable to be referred to this intervention. Clearer referral pathways for patients identified as being at risk may improve access to specialist assessment and intervention in response to screening. However, progress and outcomes of referrals made need to be monitored and incorporated into routine care planning review processes to ensure a successful intervention response when risks are identified. It was also challenging to make meaningful comparisons with international parameters (IDF, WHO, Lester UK Adaptation) as threshold criteria varied according to the comparator employed and inconsistency in data sets for age-matched populations. Standardisation of clinical assessment forms and measurement consensus for clinical markers across EIP teams may improve consistency of screening recording, and timely referrals for intervention.

## Conclusion

On presentation to an EIP service, young people with FEP are at increased risk for cardiometabolic disorders due to elevated clinical risk markers and adverse health risk behaviors. Physical health monitoring and targeted health behavior interventions, including bespoke smoking cessation, are needed early in the treatment process to attenuate identified risk markers for chronic disease and premature mortality. Streamlined processes are needed to ensure consistency in EIP service delivery and improve screening, referral and intervention processes.

## Data Availability Statement

The raw data supporting the conclusions of this article will be made available by the authors, without undue reservation.

## Ethics Statement

The studies involving human participants were reviewed and approved by University of Worcester Research Ethics Committee (REC approval no.: UWEC2014JS1). The patients/participants provided their written informed consent to participate in this study.

## Author Contributions

LG and MB devised the original protocol. DH, JS, LG and MB refined the protocol. JS was the chief investigator and oversaw the study. MB recruited participants. MB and LG were responsible for study procedures. JS and DH provided feedback on the recruitment methods and helped refine the study procedures. LG did the clinical analysis and JS oversaw the analysis. The writing team consisted of JS and LG who drafted the report manuscript. JS, LG, MB and DH were responsible for critical review of the manuscript for important intellectual content. All authors contributed to the article and approved the submitted version.

## Funding

Health Foundation Shine Award 2014.

## Conflict of Interest

The authors declare that the research was conducted in the absence of any commercial or financial relationships that could be construed as a potential conflict of interest.

## Abbreviations

EIP, Early Intervention in Psychosis; FEP, first episode psychosis; NHS, National Health Service; PA, physical activity; SMI, serious mental illness; T2DM, type 2 diabetes mellitus; MetS, metabolic syndrome.
